# Solving the transcription start site identification problem with ADAPT-CAGE: a Machine Learning algorithm for the analysis of CAGE data

**DOI:** 10.1038/s41598-020-57811-3

**Published:** 2020-01-21

**Authors:** Georgios K. Georgakilas, Nikos Perdikopanis, Artemis Hatzigeorgiou

**Affiliations:** 1 Hellenic Pasteur Institute, Athens, 11521 Greece; 2 Department of Electrical and Computer Engineering, University of Thessaly, Volos, Greece; 3 Central European Institute of Technology, Masaryk University, Kamenice 735/5, 62500 Brno, Czech Republic; 4 Department of Informatics and Telecommunications, National and Kapodistrian University of Athens, Athens, Greece

**Keywords:** Bioinformatics, Machine learning

## Abstract

Cap Analysis of Gene Expression (CAGE) has emerged as a powerful experimental technique for assisting in the identification of transcription start sites (TSSs). There is strong evidence that CAGE also identifies capping sites along various other locations of transcribed loci such as splicing byproducts, alternative isoforms and capped molecules overlapping introns and exons. We present ADAPT-CAGE, a Machine Learning framework which is trained to distinguish between CAGE signal derived from TSSs and transcriptional noise. ADAPT-CAGE provides highly accurate experimentally derived TSSs on a genome-wide scale. It has been specifically designed for flexibility and ease-of-use by only requiring aligned CAGE data and the underlying genomic sequence. When compared to existing algorithms, ADAPT-CAGE exhibits improved performance on every benchmark that we designed based on both annotation- and experimentally-driven strategies. This performance boost brings ADAPT-CAGE in the spotlight as a computational framework that is able to assist in the refinement of gene regulatory networks, the incorporation of accurate information of gene expression regulators and alternative promoter usage in both physiological and pathological conditions.

## Introduction

Cap Analysis of Gene Expression (CAGE) was initially introduced in 2003^1^ as a novel method specifically developed to capture and quantify 5′ ends of capped RNAs. During the last decade, CAGE has been continuously refined and improved into its current mature form as a well-established protocol for the identification of transcription start sites (TSS) and promoter regions of transcribed loci. The FANTOM Consortium^2^ has extensively applied CAGE on hundreds of tissues and cell lines to produce a high-quality annotation of the human and mouse promoterome and characterize regulatory mechanisms of gene expression.

Despite its increasing popularity as an experimental promoter identification protocol, the specificity of CAGE regarding the identification of transcription initiation events in the genome has several limitations. There is strong evidence^3–5^ that besides promoter regions, CAGE also identifies capping sites along various locations of transcribed loci such as different splicing products, isoforms and capped molecules that can be summed up as transcriptional noise. As a result, only a portion of regions enriched in CAGE signal were found to overlap with the surrounding region of annotated TSSs.

This poses a significant hindrance to research studies that aim to integrate regulatory regions into the framework of biological pathways. During the last decade, several *in silico* methodologies have been developed to provide basic pipelines for analyzing CAGE datasets and to facilitate peak identification and annotation. PARACLU^6^ performs clustering of CAGE tags into wider regions based on a density parameter that reflects the trade-off between size and number of overlapping reads. RECLU^7^ is an adaptation of PARACLU algorithm that is able to handle replicated (maximum two replicates) experiments based on the irreproducible discovery rate (IDR) algorithm^8^ and utilizes slightly modified parameters to filter the final results when compared against the original implementation. CAGER is the most recent *in silico* framework for CAGE analysis and TSS identification^9^. CAGER applies quality filtering, and depending on the utilized protocol, removes the 5′ end G nucleotide bias. It subsequently clusters reads into groups and is able to handle multiple CAGE experiments, detect differential TSS usage while addressing the in-between tissue variability in TSS choice and promoter shifting. The common denominator between all the aforementioned implementations is that they can be applied directly on aligned CAGE reads and do not require any additional input such as DNA sequence or other *in silico* or experimentally derived features. TSS classifier^2^, that is part of TOMETOOLS suite, was trained to distinguish between TSS and non-TSS associated CAGE signal, based on the annotation of protein coding genes. The algorithm is based on Gaussian mixture models and a random decision tree ensemble to combine their output, trained to capture the relative distribution of 4-mer occurrences in TSS and non-TSS CAGE peaks.

Despite the important advances in the development of CAGE analysis pipelines, it is evident that existing implementations still include a high number of false positive rate on TSS identification in CAGE datasets. In various older studies, unique structural features were found to be associated with promoter regions^10–13^. In a recent study^10^, the structural properties of DNA in promoter regions were investigated by analyzing 13 features including duplex disrupt energy, duplex free energy, bending stiffness, denaturation, stacking energy, bendability, propeller twist, z-DNA, A-philicity, nucleosome positioning, protein deformation, B-DNA twist and protein-DNA twist. Based on structural models derived from a wide array of biochemical experiments, sequences around transcription initiation events from DBTSS (positive set) were converted into numerical vectors and compared against their shuffled counterparts (negative set). It was shown that there are distinct patterns of structural DNA features that can distinguish between promoter and non-promoter regions. Based on these observations we hypothesized that the same features and their promoter patterns should be able to facilitate the classification of CAGE tag-clusters between real TSSs and transcriptional noise.

**Figure 1:**
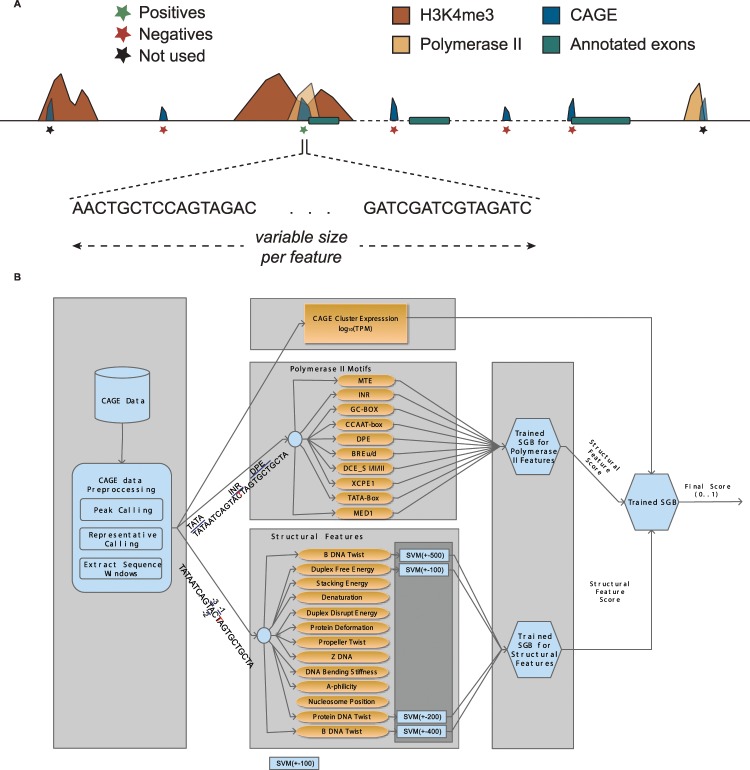
Overview of ADAPT-CAGE and the training process. (**A**) CAGE clusters selected for the positive set originated from annotated promoter regions with H3K4me3 and Polymerase II ChIP-Seq occupancy. Negative samples were selected from intronic, exonic and intergenic regions that did not overlap H3K4me3 or Polymerase II peaks. (**Β**) ADAPT-CAGE accepts as input aligned reads in BAM format. The preprocessing module aggregates CAGE reads into peaks and finds their representative nucleotide (the position exhibiting the highest expression within each peak). The sequence surrounding the representatives is extracted and the structural as well as Polymerase II features are calculated and forwarded on their own GBM (Polymerase II motifs) and SVM (structural features) models. The last module of the framework is based on a GBM model that combines previous outputs with the expression level of candidate TSSs to produce the final output of the algorithm.

In this study, we present ADAPT-CAGE, a versatile computational framework for analyzing CAGE data providing highly accurate experimentally derived TSSs on a genome-wide scale. The proposed algorithm (Fig. 1) introduces a novel approach for distinguishing CAGE tag-clusters that represent transcription initiation events from clusters that are formulated due to recapping events, byproducts of the splicing machinery as well as transcriptional and/or sequencing noise. ADAPT-CAGE can be directly applied to aligned CAGE reads, however, in contrast to existing implementations, it is also based on structural and promoter-associated motif features extracted from the underlying DNA sequence. Initially, CAGE tags are filtered based on their mapping quality and 5′ ends of the remaining reads are combined into peaks if they are located closer than a user-specified number of base pairs. Peaks are further filtered if the expression level is lower than a cutoff defined by the user. For each CAGE peak, the normalized number of overlapping reads is computed and the representative nucleotide is selected based on the localization of CAGE tag 5′ ends. The algorithm strategically utilizes sequence and structural DNA properties that have been previously^10^ associated with transcription initiation and Polymerase II promoter regions (Supplementary Fig. 1). Based on the sequence surrounding each representative TSS, ADAPT-CAGE extracts sequence and structural DNA features which are then forwarded into a multilayered Machine Learning module. This module is based on Support Vector Machines (SVM) and Stochastic Gradient Boosting (SGB) models individually trained on each DNA feature and subsequently combined with an agent assembly strategy.

## Results

### Genome-wide assessment of FANTOM CAGE tag cluster quality, pre- and post-ADAPT-CAGE application

There is increasing evidence in the literature^5,14^ that unveils CAGE’s property to detect recapping events, alternative isoforms and in some cases, post-transcriptional processing and cleavage by RNA binding proteins, in addition to transcription initiation events. To showcase the aforementioned properties, we adopted^15^ an unsupervised exploratory strategy on a genome-wide scale utilizing ChIP-Seq data against H3K4me3, H3K4me1, H3K27ac, H3K27me3, H3K9me3 and H3K36me3. These histone marks are traditionally used to characterize chromatin states in landmark studies and projects such as Roadmap Epigenomics^16^. The idea behind this approach is to group CAGE enriched regions based on the surrounding chromatin state, before and after the application of ADAPT-CAGE, aiming to highlight the noise embedded into raw CAGE signal and the algorithm’s ability to effectively remove it.

**Figure 2:**
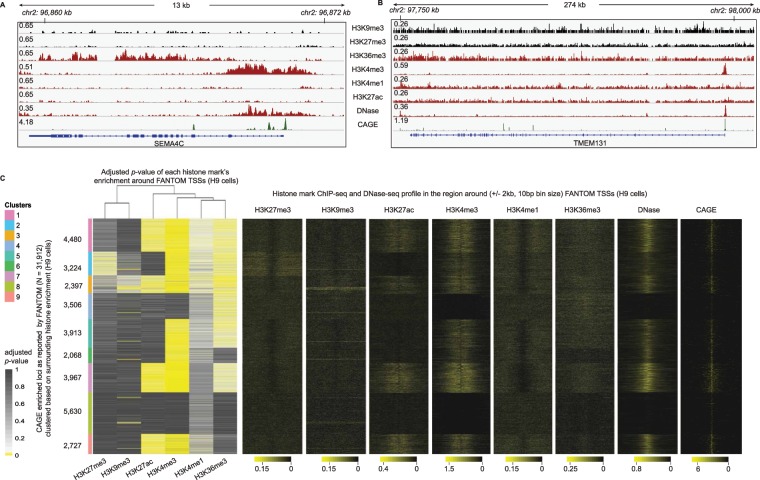
Genome-wide analysis of the chromatin landscape surrounding CAGE enriched loci in H9 cells as reported by FANTOM. Examples of (**Α**) SEMA4C and (**Β**) TMEM131 loci with tracks from six histone marks showing chromatin status, DNase-Seq unveiling accessibility and the raw CAGE signal. Active or poised promoters are denoted with H3K4me3 and H3K27ac, active or poised enhancers with H3K4me1 and H3K27ac, repressed chromatin with H3K27me3 and H3K9me3, and transcribed regions with H3K36me3. As indicated by the CAGE signal, there are multiple CAGE enriched regions located in introns and exons of SEMA4C and especially of TMEM131 with length over 200 kb. The ones located in promoter regions exhibit increased chromatin accessibility while the rest are located in inaccessible chromatin. (**C**) 31,912 FANTOM CAGE peaks were clustered based on the enrichment of six histone marks (H3K4me3, H3K27ac, H3K4me1, H3K36me3, H3K9me3 and H3K27me3). On the right side, histone ChIP-, DNase-Seq and CAGE signal profile is also shown. We observe that 61.3% (19,552) of CAGE peaks exhibit active histone mark enrichment and the remaining 38.7% (12,360) do not demonstrate any chromatin activity showcasing the increased transcriptional noise level embedded in CAGE datasets.

To this end, 65,141 CAGE peaks/tag-clusters in H9 cells were extracted from FANTOM repository. Tag-clusters located closely to one-another (less than 1 kb), thus belonging in the same promoter region, were merged into a unique event. The position with the highest coverage of reads was selected as a representative, resulting in 31,912 transcription initiation events. The occupancy of each histone mark was calculated in their surrounding region (+/−1 kb). The histone mark enrichment formed the basis for clustering these regions into groups with similar patterns of chromatin activity (Fig. 2). Normalized signal profiles were also added as a visual aid. In Fig. 2A,B, two active loci (SEMA4C and TMEM131) are shown along with the normalized CAGE, ChIP- and DNase-Seq signal depicting different types of activity. In both cases, DNase-Seq and H3K4me3 ChIP-Seq data indicate that both promoters are accessible and CAGE shows that the two genes are expressed. We can observe numerous CAGE enriched regions in their exons and introns, especially in the case of TMEM131 whose length is above 200 kb.

Similar activity patterns are also observed on the genome-wide scale (Fig. 2C). There are two major chromatin modes around CAGE enriched loci. Most of them (61.3%, 19,552) exhibit, as expected, a medium to strong enrichment in DNase- and histone marks ChIP-Seq related to active promoters/enhancers. The remaining loci (38,7%, 12,360) in clusters 2, 4 and 8, do not exhibit enrichment in DNase-seq signal nor in any of the histone marks. Evidently, the members of cluster 2, 4 and 8 represent the previously described noise embedded into raw CAGE signal, since the activity of these genomic regions is not supported by chromatin activity related experimental data. These findings are in tandem with the evidence from the literature, highlighting the significant enrichment of CAGE datasets in signal that corresponds to transcriptional noise.

The question that arises is how the picture of the chromatin landscape around the aforementioned CAGE enriched regions changed after applying ADAPT-CAGE. The last layer of the ADAPT-CAGE architecture consists of an SGB model that provides a probabilistic final output score (Fig. 1B). Based on the probabilistic nature of ADAPT-CAGE’s final score, we considered 0.5 as a reasonable (loose) cutoff for generating positive and negative predictions. From the initial set of regions (N = 31,912), 17,850 loci passed (Supplementary Fig. 2A) and 14,062 were scored below the cutoff (Supplementary Fig. 2B). The vast majority (97.1%, 17,335 out of 17,850) of the positively scored CAGE enriched regions exhibit medium or strong enrichment in histone modifications signal of active transcription. Cluster 9 consisting of only 515 regions is the only group that is not enriched in such signal. In contrast, 9,624 out of 14,062 (clusters 2, 5, 7, 8, 9 and 10 in Supplementary Fig. 2B) loci that failed to pass the ADAPT-CAGE score cutoff do not exhibit any enrichment in active transcription signal, while the remaining 4,438 presents medium to strong activity. We also applied a more stringent score cutoff (0.9) that is related to the desired precision/sensitivity balance for generating positive (Supplementary Fig. 3A) and negative (Supplementary Fig. 3B) predictions.

The evidence presented in this section support that ADAPT-CAGE is able to correctly distinguish between CAGE enriched regions associated to transcription initiation events from noise. However, even though ADAPT-CAGE exhibits very high levels of precision, the performance in terms of sensitivity seems to be lagging behind in this analysis. In general, every unsupervised learning strategy inherently produces results with increased within-cluster variability and k-means is not the optimal algorithm for dealing with non-linearity. In addition, the lower levels of sensitivity could also be explained by the limitations of the experimental methods used to produce the genome-wide histone occupancy and the inability to detect lowly expressed regions due to the selected sequencing depth. In any case, the unsupervised strategy presented here provides only a rough estimate of ADAPT-CAGE’s performance. In the following sections, we present more results from benchmarking strategies based on additional experimental evidence and the annotation of the human genome as well as performance comparisons with existing algorithms. The number of total predictions with default settings for each algorithm are listed in Supplementary Table 1. For the remaining evaluation approaches, an ADAPT-CAGE score cutoff of 0.9 was utilized, unless stated otherwise (i.e. the application of multiple score cutoffs for generating curves). The threshold of 0.9 reflects the desired balance between precision and sensitivity (data not shown).

### Experimentally- and ChromHMM-driven comparison between ADAPT-CAGE and existing algorithms

The Roadmap Epigenomics^16^ and ENCODE^17^ projects have long been a one-stop data repository for epigenomics and transcriptomics providing hundreds of high quality samples from multiple tissues and cell lines. This wealth of information enabled the development of numerous computational methods such as ChromHMM^18^ which has emerged as a powerful approach for annotating the epigenome based on experimentally-driven identification of cell-specific chromatin states.

To further explore the limits of ADAPT-CAGE’s performance and compare it with existing algorithms, we developed a benchmarking strategy based on the core-15 chromatin states from ChromHMM (Supplementary Table 2) in H9 and K562 cells. This evaluation process shares some properties with the unsupervised approach described in the previous section, since both of them rely on epigenetic experimental data. However, with this analysis, we aim to provide an alternative epigenome-driven evaluation that is based on a popular Machine Learning algorithm, such as ChromHMM, that is considered as the golden standard method for annotating epigenomes.

**Figure 3:**
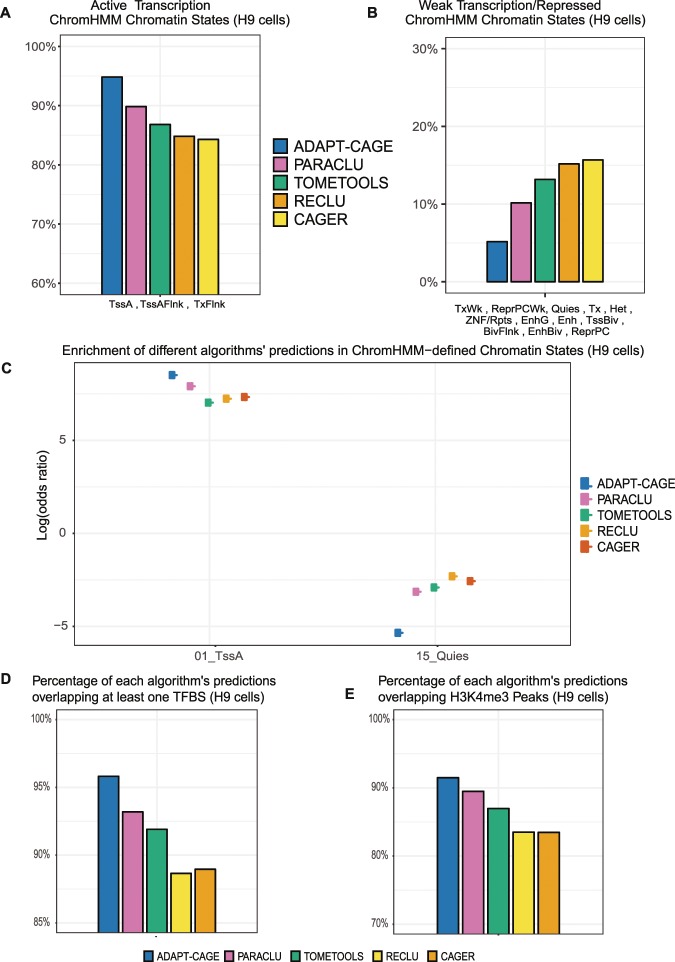
Evaluating algorithms’ performance on experimental data in H9 cells. Percentage of each algorithm’s predictions uniquely overlapping ChromHMM-derived chromatin states based on the core-15 model in H9 cells. Chromatin states were aggregated in two groups according to levels of chromatin/transcriptional activity. Active (**A**) and weaker (**B**) transcription states. (**C**) Enrichment (odds ratio in logarithmic scale) of algorithms’ prediction in the two most orthogonal ChromHMM-derived chromatin states (TssA and Quies). Percentage of each algorithm’s predictions overlapping at least one transcription factor binding site (**D**) and H3K4me3 (**E**) ChIP-Seq derived peaks.

Initially, for every algorithm we calculated the percentage of predictions that overlap each chromatin state (Fig. 3A,B, Supplementary Fig. 4A,B and Table 3). When a prediction overlapped multiple states, we assigned it to the state with which exhibits the highest overlap. The chromatin states were aggregated into two groups based on their relation with active or repressed chromatin. In the active chromatin group (TssA, TssAFlnk and TxFlnk), the top-performing algorithm is ADAPT-CAGE (94.85%) followed by PARACLU (90.5%), TOMETOOLS (86.98%), RECLU (85.22%) and CAGER (84.66%). ADAPT-CAGE also exhibits the lowest percentage in the repressed chromatin group (5.12%), followed by PARACLU (9.36%), TOMETOOLS (12.96%), RECLU (14.59%) and CAGER (15.12%). The percentages from the two categories of chromatin states do not add up to 100%, as expected, because there are a few predictions per algorithm that do not overlap any ChromHMM-annotated part of the genome. The results for the K562 comparison are shown in Supplementary Fig. 4A,B and all data can be found in Supplementary Table 3.

To compare the performance in a more unbiased way that alleviates the different number of predictions provided by each algorithm, we proceeded with the calculation of the odds ratio (OR) for the enrichment of positively scored predictions in each chromatin state, in logarithmic scale (Fig. 3C). The OR for each algorithm in the two most orthogonal, in terms of activity, states is shown. TssA state characterises active TSS-proximal promoters and Quies state describes regions with no histone enrichment. In both cases ADAPT-CAGE outperforms all other algorithms. The results for the K562 comparison are shown in Supplementary Fig. 4C.

To further explore the algorithms’ performance without using any third-party computational method, transcription factor binding sites (TFBS) were downloaded from the ENCODE Txn factor track that incorporates ChIP-Seq derived binding sites for more than 160 transcription factors. The overlap of each algorithm’s prediction with at least one TFBS in H9 cell lines was calculated (Fig. 3D). ADAPT-CAGE achieves the best performance (95.81%) followed by PARACLU (93.19%), TOMETOOLS (91.9%), CAGER (88.96%) and RECLU (88.65%). Similar results are shown in Supplementary Fig. 4D for the same comparison based on K562 data (Supplementary Table 3).

The last benchmark in the experimentally oriented group of comparisons was based on peaks derived from ChIP-Seq data against H3K4me3, a histone modification that is highly enriched at active promoters (Fig. 3E). CAGE enriched regions that were positively scored by ADAPT-CAGE demonstrate the highest overlap with H3K4me3 peaks (91.48%), followed by PARACLU (89.48%), TOMETOOLS (86.96%), RECLU (83.49%) and CAGER (83.47%). Similar observations were made in the comparison based on K562 data (Supplementary Fig. 4E and Table 3).

### Hybrid approach for comparing ADAPT-CAGE and existing algorithms

The evaluation presented in the previous section was based on the output of ChromHMM, which utilizes histone mark ChIP-Seq data to annotate the genome with distinct cell-specific chromatin states. One limitation of this approach is related to the data quality and sequencing depth, parameters that might affect the accurate assignment of lowly expressed promoters to active chromatin. We sought to perform a more systematic evaluation of algorithms, one that relies on RefSeq protein coding gene annotation and H3K4me3 ChIP-Seq from H9 cells.

**Figure 4:**
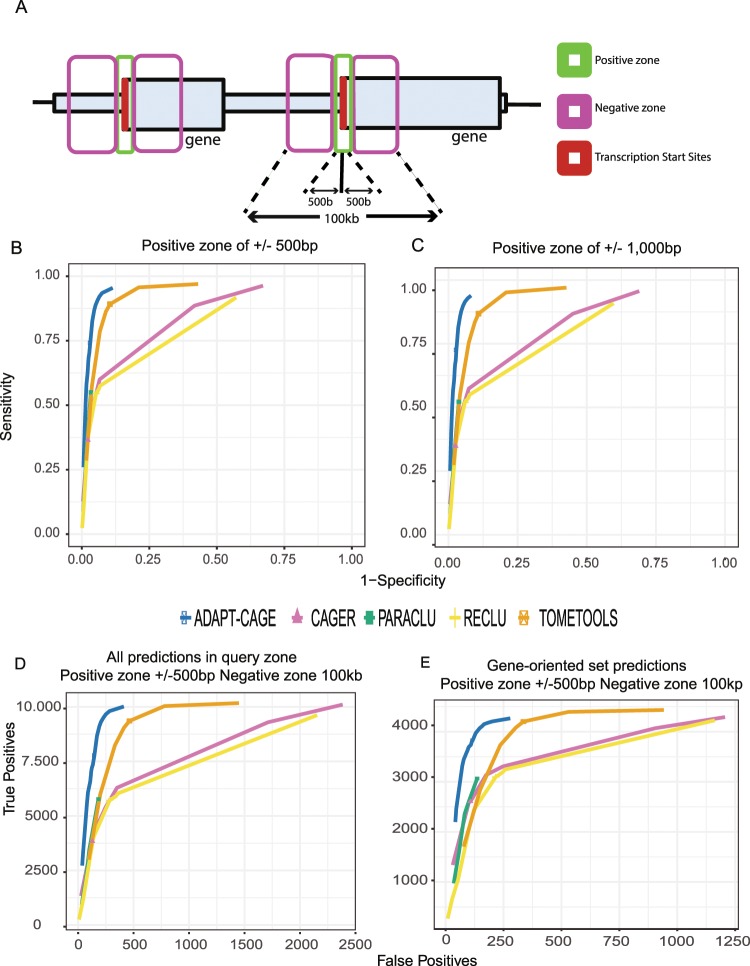
Evaluating algorithms’ performance in H9 cells based on the combination of the human genome annotation and H9 H3K4me3 ChIP-Seq data. For a more systematic comparison of the algorithms’ performance, the human genome annotation was utilized for generating positive and negative zones surrounding annotated protein coding TSSs (**A**). The positive zone was defined as a +/−500 bp window centered on annotated TSSs. Predictions in this zone were accepted as positives. In the remaining region outside this zone and up to +/−50 kb away from TSSs (negative zone), all predictions were accepted as negatives, except for predictions that overlapped with H3K4me3 ChIP-Seq peaks that were considered positives. ROC curves based on two different positive zone sizes, +/−500 bp (**B**) and +/−1 kb (**C**). The curves were generated by applying multiple ADAPT-CAGE and TOMETOOLS score cutoffs and a range of different parameters for the application of remaining algorithms (from loose to stricter outcomes). The complete range of utilized parameters are shown in Supplementary Table 4. Number of true positives plotted against false positives based on the +/−500 bp positive zone (**D**), and based on a gene-oriented strategy (**E**). In (**E**), a true positive prediction is a gene that has at least one prediction in the positive zone (+/−500 bp around its TSS) and a false positive prediction is a gene with at least one prediction in the negative zone but no predictions in the positive zone.

The region surrounding annotated TSSs (+/−50 kb) were selected to form the set of benchmark parts of the genome (see Materials and Methods). Initially, all CAGE peaks located +/−500 bp around TSSs were considered as positive, while the ones located in the remaining parts of the benchmark regions were treated as negative (Fig. 4A). The same strategy has previously been used in^2^. In the second approach, we expanded the positive zone to +/−1 kb. CAGE peaks located in the negative zones that overlapped with H3K4me3 ChIP-Seq peaks were treated as positive instead of negative. This definition of positive and negative zones enabled the calculation of performance metrics such as specificity and sensitivity in H9 cell line. This hybrid strategy is able to remove certain obstacles that may arise in an annotation-only based evaluation. The main disadvantage is related to alternative promoter usage. Alternative promoters might frequently be located outside of the loci selected as positive zones, severely affecting the comparison results. The incorporation of H3K4me3 ChIP-Seq peaks in the process of generating the positives alleviates such effects.

In contrast to other algorithms, ADAPT-CAGE and TOMETOOLS are the only methods that provide a score to every processed CAGE enriched region. Therefore, by applying multiple score cutoffs, we evaluated ADAPT-CAGE and TOMETOOLS performance at each threshold. To perform a similar evaluation on the remaining algorithms, we applied them using different parameters at each run (Supplementary Table 4), thus creating a set of different results (from loose to stricter outcomes). We observe that ADAPT-CAGE outperforms all other algorithms in both +/−500 bp (Fig. 4B) and +/−1,000 bp (Fig. 4C) positive zone strategies (Supplementary Table 5). In Fig. 4D we present the results based on the +/−500 bp positive zone in absolute numbers of true positives (TPs) and false positives (FPs).

The previous benchmark was a CAGE peak centric approach. For the following comparison we adopted a gene oriented strategy. A positive prediction is a gene that has at least one hit in the +/−500 bp region around its TSS. The results are shown in Fig. 4E depicting that ADAPT-CAGE outperforms all existing algorithms (Supplementary Table 6).

As a final comparison of algorithms in H9 cells, we counted the overlap of positively scored CAGE enriched loci with promoters, introns, exons and splicing sites (Supplementary Fig. 6A). The total amount of predictions of each algorithm, in this evaluation, corresponds to the number of predictions overlapping the aforementioned genic annotation categories. Predictions located in other parts of the genome were not considered. ADAPT-CAGE exhibits the highest overlap with promoters (92.73%), followed by PARACLU (88.35%), TOMETOOLS (85.74%), RECLU (83.12%) and CAGER (82.47%) as shown in Supplementary Fig. 6B and Table 7. Additionally, ADAPT-CAGE presents the lowest overlap with other parts of genic loci, except introns (Supplementary Fig. 6C).

## Discussion

Eukaryotic organisms exhibit diverse gene expression patterns, a feature that is responsible for the spatio-temporal cell diversity in such multicellular species. Unlocking the mechanisms that regulate transcription is a fundamental step towards understanding the rules of biological systems that when disrupted, diseases emerge. To achieve this, easy-to-implement experimental protocols and robust computational solutions are imperative. With the advent of Next Generation Sequencing, experimental methods able to quantify gene expression and/or transcript abundance have emerged. CAGE is one of these approaches.

CAGE was initially introduced in 2006 and since then it has been continuously refined and improved into its current, significantly more mature, status. Nowadays, CAGE is considered a state-of-the-art protocol for experimental identification of transcription initiation events. Despite its popularity, many studies^3–5,14^ have unveiled that CAGE is able to identify a wide array of biological events besides transcription initiation such as byproducts of the splicing machinery and transcriptional noise in general. Naturally, these observations suggest a significant number of false positive TSSs identified in CAGE datasets. In this study, we unveiled this problem on a genome-wide scale, using an unbiased approach that involved unsupervised learning and epigenetic datasets (Fig. 2). More than one third of the H9 CAGE tag-clusters provided in FANTOM database are located in repressed chromatin exhibiting zero transcriptional activity.

This poses a significant obstacle to transcription-related research, signifying the need to develop computational methods for excluding events captured with CAGE that do not associate with TSSs. There are a few *in silico* frameworks, TOMETOOLS, PARACLU, RECLU and CAGER, that have been designed specifically to process raw CAGE data and identify regions of the genome that are enriched in CAGE signal. PARACLU is an algorithm that clusters reads to form peaks. This is also true, in a sense, for RECLU and CAGER since both of them use PARACLU (CAGER can also use distclu) as their first step and then apply filters of statistical nature among others. Besides TOMETOOLS, there is no Machine Learning involved in the pipeline of the aforementioned algorithms. However, the evidence in the literature shows that strong CAGE enrichment can also be found in non-promoter regions, therefore there is not a one-to-one association between high read-count CAGE peaks and transcription initiation events. That was exactly the type of evidence which lead to the development of ADAPT-CAGE and the application of Machine Learning for distinguishing between CAGE enriched regions that belong into the transcription initiation event class and other events or sequencing noise.

Evaluating and comparing the performance of algorithms with different properties and mechanisms is not a straightforward task. Our strategy was focused on generating benchmarking datasets that are unbiased against one algorithm or the other, as much as possible. To this end, the evaluation process involved, epigenetic experimental data indicative of active transcription, third-party Machine Learning software for annotating chromatin states, coverage in TF binding events and different combinations of protein coding gene related annotations.

In truth, every evaluation approach offers certain advantages and disadvantages that, to some extent, might affect the comparison of algorithms. One of the disadvantages, in the annotated TSS based evaluation for example, is the definition of positive (+/−500 bp around the TSS) and negative zones (+/−50 kb around the TSS, excluding the positive zone). In this scenario, there is a possibility of alternative promoters being expressed in the selected negative zones, therefore affecting the comparison results. In reality, we will never be able to fully correct for such problems, and this is a fundamental issue that exists in every annotation-based evaluation approach. The pace of discovering new functional regions in the genome is set too high by Next Generation Sequencing, however the incorporation of de novo elements into the genome annotation is severely lagging behind. We attempted to alleviate the effects of this problem by following a hybrid strategy and combining protein coding gene annotation and experimental data derived from H3K4me3 ChIP-Seq.

Despite all the obstacles described above, we believe that every type of evaluation is able to unveil properties of the tested algorithms that other approaches cannot. In fact, there are caveats in every evaluation strategy, and for this reason we decided to apply multiple approaches, attempting to complement the disadvantages of one strategy with the advantages of another.

Several observations were made regarding existing algorithms, based on the evaluation results. Specifically, most algorithms involve stringent cutoffs in their pipeline resulting in the removal of valuable information from the CAGE tag-cluster set. The remaining tag-clusters exhibit high coverage in CAGE reads but that does not imply they are true positive transcription initiation events. Interestingly, PARACLU is performing better than the other existing implementations, despite its simplicity. On the other hand, ADAPT-CAGE is more sensitive and harnesses the power of Machine Learning on promoter-associated and structural DNA features to provide highly accurate predictions without removing the majority of candidate TSSs. It is able to perfectly complement research oriented around transcriptional mechanics by removing CAGE tag-clusters or genomic loci that are not associated with true TSSs.

In light of Next Generation Sequencing, accurate computational methods such as ADAPT-CAGE can emerge as key components of studies that aim to identify the regulatory machinery and core constituents of biological pathways. This is a fundamental endeavor towards understanding and characterizing the underlying mechanisms that govern every aspect of physiological conditions in biological systems, as well as stimuli that cause the transition into pathological states.

## Materials and Methods

### CAGE- and ChIP-Seq data analysis and utilized annotation

For the training and evaluation process we used CAGE samples already pre-processed and aligned on GRCh38 assembly by FANTOM. There are multiple ways to retrieve information from FANTOM repository, ranging from sam formatted alignments per sample to files consolidating all of the identified CAGE enriched loci with expression levels across the entire panel of profiled cell types. ADAPT-CAGE provides two modes for utilizing CAGE information in the algorithm (see Overview of ADAPT-CAGE section). Briefly, the first mode starts from CAGE sample bam files, creates peaks and identifies representative nucleotides for each peak. Subsequent steps of the algorithm are then based on these representatives. The second mode starts from bed formatted files of already identified peak representatives as specified by the user.

The training of ADAPT-CAGE (see the relevant section for more details) was based on the aforementioned first mode of utilizing CAGE information that was applied on CAGE bam files from H1 cells with FANTOM ids CNhs14067, CNhs14068 and CNhs13964. For the evaluation process we used CAGE samples from H9 and K562 cells with FANTOM ids CNhs11917, CNhs12824, CNhs12824, CNhs12458, CNhs12684 and CNhs12786^2^. The file named “hg38.fair + new_CAGE_peaks_phase1and2_tpm.osc.txt.gz”, which contains all characterized CAGE peaks (N = 211,744) and their expression on all cell types, was downloaded from FANTOM repository alongside CAGE tag start site (CTSS) bed files related to these samples. The file including all identified CAGE peaks was filtered based on the expression level of peaks (tpm > 0) in H9 and K562 sample ids to create two sets of peaks (Supplementary Table 1), one for each cell type (65,141 peaks in H9 cells and 47,377 in K562). These sets were combined with the CTSS list to generate peak representatives, on which ADAPT-CAGE was applied, to provide predictions that participate in all evaluation results. The CTSS bed files were converted to the appropriate format required by CAGER, RECLU and PARACLU. Bam files for the H9 samples were also downloaded to calculate the average profile of CAGE signal shown in Fig. 2 and Supplementary Figs. 2 and 3.

ChIP-Seq datasets in the aforementioned cell-lines were obtained from the ENCODE repository (specific ENCODE sample ids are shown in the following sections). NCBI RefSeq database^19^ has been utilized as the reference annotation of coding and non-coding genes that were incorporated in the process of training the ADAPT-CAGE modules and evaluating all algorithms. For the TFBS-oriented evaluation, the genomic coordinates for TFBSs were downloaded from the ENCODE ‘Txn Factor’ track. This track incorporates a large collection of ChIP-Seq derived TFBSs from 161 TFs in 91 cell types.

The epigenomic data evaluation was based on the ChromHMM 15-states annotation (Supplementary Table 2) obtained from the Human Epigenomics^16^ repository. E008_15_coreMarks for H9 and E123_15_coreMarks for K562 were downloaded and converted to GRCh38 compatible coordinates. In case of predictions overlapping multiple chromatin states, we assigned the CAGE enriched region to the state with which exhibits the highest overlap. Additionally, H3K4me3 ChIP-Seq derived peaks with sample ids ENCSR716ZJH and ENCSR000EWA for H9 and K562 cells were used.

The hybrid evaluation was based on RefSeq protein coding gene annotation and H3K4me3 ChIP-Seq data in H9 cells. We defined positive and negative zones around annotated TSSs in a two-fold strategy (Fig. 4A). In the first one, the positive zone was defined as a +/−500 bp window centered on annotated TSSs and the negative zone as a +/−50 kb window centered on same TSSs excluding the positive zone (Fig. 4B). For the second strategy, the positive zone was extended to +/−1 kb (Fig. 4C). The caveat with this type of evaluation is related to alternative promoters. This type of promoters might be expressed in the profiled cell types while being located in the negative zone. To correct for such cases as much as possible, regions embedded into the negative zones that overlapped with H3K4me3 ChIP-Seq peaks were treated as positive zones instead of negative.

### Overview of ADAPT-CAGE

The implementation of ADAPT-CAGE is based on a modular approach layering a series of ML models (Fig. 1). It takes advantage of the difference of structural DNA features and Polymerase II associated motifs between promoter and non-promoter regions (Supplementary Figs. 1 and 5). The algorithm initially processes aligned CAGE reads to identify loci/peaks enriched in CAGE signal. Reads are filtered by a user-defined mapping quality threshold (default is 10) and the 5′ ends of remaining reads are combined into peaks if they are located closer than a number of base pairs specified by the user (default is 50 bp). Peaks with normalized read-count less than a user-defined cutoff (default is 0.5 tpm) are discarded. The nucleotide exhibiting the highest number of overlapping 5′ tag ends is selected as representative for subsequent analysis. Alternatively, ADAPT-CAGE also accepts bed formatted files with coordinates of already defined CAGE tag-cluster representatives. Windows of varying size are extracted, centered on the representative nucleotide and vectorized using various structural feature characteristics. Each vector is forwarded into a uniquely trained SVM model to obtain a single probabilistic value representing its significance. A specifically trained Stochastic Gradient Boosting (SGB) model integrates the output values of all SVM models and emits the combined importance of the structural feature set. The affinity of Polymerase II associated motifs is assessed through 100 bp windows around each CAGE peak representative. Polymerase II motifs is integrated into a separately trained SGB model that provides the combined significance of these DNA patterns. Τhe final step of the pipeline, incorporates the structural and Polymerase II motifs with the normalized expression level of each CAGE tag-cluster representative into a final SGB model that distinguishes between transcription initiation events and noise.

### Feature analysis of training dataset

For the quantitative analysis of structural features the sequence around CAGE tag-cluster representatives was converted into numerical profiles (Supplementary Fig. 5). Each di- or tri-nucleotide was assigned a value derived from extensive biochemical studies summarized in^20^. A sliding window was subsequently applied to smooth the raw profiles.

Following previous studies^10–13^, we analyzed the profile of structural DNA features to observe the aggregated differences between the positive and negative set of CAGE peaks (see the first section in Materials and Methods for the creation of positives and negatives). As expected, similar patterns were observed for the positive set of CAGE tag-clusters compared to the negative set. One characteristic example of A-philicity is shown in Supplementary Fig. 1. Furthermore, we analyzed the profile of thirteen Polymerase II associated motifs (Supplementary Fig. 1D) derived from JASPAR database^21^. These motifs represent various promoter-related elements such as Inr, TATA-box, MTE, GC-Box, CCAAT-Box, DPE, BREu, BREd, DCE-S-I, DCE-S-II, DCE-S-III, XCPE1 and MED1. In this case, we also observed striking differences between the positive and negative set. Supplementary Fig. 1B shows the INR motif distribution on different categories of said data.

The importance of each feature was assessed with the feature selection module of Boruta^22^ package in R (Supplementary Fig. 1C,D). The importance measure of each feature was obtained as the loss of accuracy of classification caused by the random permutation of attribute values between objects. The importance measure (Y-axis) itself varies due to the usage of different data sets for the Polymerase II and structural features. It also depends on the inherent stochasticity of the random forest classifier as well as the presence of non important attributes in the information system (shadow features)^22,23^.

The performance of each structural feature fluctuated depending on the combination of the queried sequence size (window around CAGE tag-cluster representatives) and the smoothing parameter (sliding window size). The Polymerase II motif affinity was calculated using TRAP^24^ and the weight matrices from the JASPAR database. The best performing window surrounding the CAGE tag-cluster representatives was found to be of 100 nucleotides in size (data not shown). Details regarding the models’ training are presented in the following paragraphs.

### ADAPT-CAGE training

CAGE reads, from H1 cells, with less than 10 mapping quality were removed. Reads were merged into peaks/tag-clusters if they were located closer than 50 bp. From the initial set of tag-clusters only those exhibiting a normalized expression level of more than 0.5 tpm were retained for downstream analysis (Supplementary Fig. 5). Tag-clusters located closer than 500 bp from annotated TSSs and exhibiting an overlap with H3K4me3 and Polymerase II ChIP-Seq peaks consisted the positive set. Tag-clusters overlapping intronic and exonic regions or located in intergenic space, but not overlapping H3K4me3 or Polymerase II peaks, consisted the negative set. The resulting set of 16,573 (7,614 positives and 8,959 negatives) tag-clusters was divided into 4 non-overlapping subsets consisting of three training sets and one test set for evaluating different combinations of ADAPT-CAGE sub-models. The first training set (3,807 positives and 4,480 negatives) was utilized to train thirteen distinct SVM models (with GPU acceleration) using libsvm^25^, one for each structural DNA feature, as well as for training the SGB model for the Polymerase II associated motifs. Even though SVM models are theoretically resistant to the curse of dimensionality, the ratio of training samples versus sample dimension was approximately 6:1 to avoid any potential overfitting. Additionally, 10-fold cross validation (CV) was applied during the training process to optimize the generalisation properties of the final models. The second training set (1,524 positives and 1,793 negatives) formed the basis for training the SGB model that combines the results of the individual SVM models related to the structural DNA features. The third training set (1,143 positives and 1,344 negatives) was utilized to train the final SGB model that combines the output of the last tier of SGB models plus the normalized expression level in log_10_ scale. The final set that served as our benchmark consisted of 1,140 positives and 1,342 negatives. Supplementary Fig. 5 demonstrates the different sets used for ADAPT-CAGE training and benchmarking.

The assessment of the importance of the final structural and promoter feature models, as well as the normalized expression level for the classification task was performed with Boruta R package (Supplementary Fig. 1E). The training of all SGB models was performed with caret package in R^23^, using 50 iterations for parameter tuning, and 10-fold CV repeated 10 times to estimate performance and avoid overfitting. SGB was compared against typical Random Forests, Boruta Random Forests, SVM, Self-Organizing Maps and Multilayered Perceptrons and was found to be the best performing algorithm (data not shown).

### Clustering CAGE derived TSSs based on histone mark enrichment

For the purposes of this analysis, we further processed the 65,141 CAGE peaks/tag-clusters in H9 cells that have been described in a previous section. To eliminate the possibility of incorporating CAGE tag-clusters located closely to one-another, thus belonging in the same promoter region, peaks with a distance less than 1 kb were merged into a unique event, considering strandness. For each merged event, the position/nucleotide with the highest coverage of CAGE tags was chosen as a representative. This resulted in 31,912 unique transcription initiation events. The whole rationale of this study is that when it comes to utilizing CAGE as a TSS identification methodology, it can be quite noisy. This observation has repeatedly been reported in the literature, and in Fig. 2A,B we are able to visually inspect this issue in two randomly chosen loci. To assess the severity of the problem genome-wide, we thought of using histone mark ChIP-Seq data in H9 cells from ENCODE, and cluster the 31,912 unique TSS event into groups of similar epigenetic states^15^. To this end, the enrichment of H3K4me3 (ENCFF161EGP ENCODE id), H3K4me1 (ENCFF804YUX), H3K9me3 (ENCFF049TFL), H3K27me3 (ENCFF022SFF), H3K36me3 (ENCFF760NOJ) and H3K27ac (ENCFF271XBU) versus Input (ENCFF734TEC) in the +/−1 kb region surrounding CAGE tag-cluster representatives has been calculated with normR^26^. These enrichment results facilitated the k-means clustering of CAGE tag-clusters in 9 groups, following silhouette coefficient analysis (data not shown). The adjusted *p*-value was used as a proxy of the enrichment of each mark, to cluster CAGE peaks with similar chromatin states. For visualization purposes, the normalized histone mark ChIP-Seq as well as DNase-Seq (ENCFF110SPC) enrichment was also calculated for 10 bp non-overlapping bins spanning the +/−2 kb region centered on CAGE tag-cluster representatives. The same analysis was performed after applying ADAPT-CAGE with a score cutoff of 0.5 to the set of 31,912 CAGE enriched loci, separately to the positively (17,850 loci grouped into 10 clusters) and negatively (14,062 loci also grouped into 10 clusters) scored regions (Supplementary Fig. 2A,B). We also applied a more stringent cutoff (0.9) and applied the same technique to the positively (15,029 loci grouped into 10 clusters) and negatively (16,883 loci also grouped into 10 clusters) scored regions (Supplementary Fig. 3A,B).

### Genomic location-based evaluation

For the genomic location-based evaluation we considered the overlap of each algorithm’s prediction set with subparts of genic loci (introns, exons and splicing sites) as well as promoter regions (Supplementary Fig. 6A). The total amount of predictions of each algorithm, in this evaluation, corresponds to the number of predictions overlapping the aforementioned genic annotation categories, while predictions located in other parts of the genome were discarded. In view of the fact that many predictions can overlap more than one type of annotation, we defined a hierarchy of location:prediction assignments. For instance, a prediction located 100 bp downstream from a TSS could overlap a promoter and the first exon. Another example could be that candidate TSS events might overlap intronic/exonic regions on a cell-type dependent splice processing. For this reason, the annotation category assignment of candidate TSSs was prioritized with the following strategy (even if the width of the overlapping region is one base pair). Promoter regions (1,000 bp upstream and 500 bp downstream of an annotated TSS) had the highest priority, followed by exon/intron junction regions (+/−50 bp around annotated splice sites). Lastly, exons and introns exhibited the lowest priority.

### Application of existing implementations

One of the differences between ADAPT-CAGE and other implementations, besides TOMETOOLS, is that it assigns a probabilistic score to each peak. Therefore, by applying several score cutoffs (Supplementary Table 4), we managed to explore the complete range of ADAPT-CAGE and TOMETOOLS performance. To achieve the same type of evaluation for PARACLU, RECLU and CAGER, we applied them multiple times, using different parameters at each run (Supplementary Table 4), thus creating several sets of results corresponding to distinct strictness levels.

PARACLU was applied with “num of reads” parameter values of 30, 50, 60 and 90 and “density” values of 2, 4, 8, 16, 32, 320 and 1000. RECLU was applied with “tpm” values of 0.1, 0.5, 1, 5, 10 and 30 and “IDR” values of 0.001, 0.05, 0.1, 0.5 and 1. CAGER was applied with “tpm” values of 0.1, 0.5, 1, 10 and 50, “method” values of PARACLU and distclu and “maxDist” values of 1, 10 and 20.

We have applied all algorithms with every combination of parameters but we only show results for a subset of combinations (Supplementary Table 4) for which we observed differences in the output. For PARACLU, “num of reads” was kept at 30 and “density” values were 2, 32, 320 and 1000. Regarding RECLU, for “IDR” of 0.1 we selected “tpm” values of 0.1, 0.5, 1, 5, 10 and 30 and for “tpm” of 0.1 we selected “IDR” values of 0.001, 0.05, 0.1, 0.5 and 1. In CAGER, “method” was PARACLU and for “maxDist” of 20 we selected “tpm” values of 0.1, 0.5, 1, 10 and 50 and for “tpm” of 1 we selected “maxDist” values of 1 and 10. The default values of these parameters are also included in the selected ranges and they are depicted with specific shapes (triangle, square etc) in Fig. 4. For the results of the comparisons shown in the remaining figures, the output of algorithms using default parameter values was used.

Specifically for the hybrid comparison presented in Fig. 4B,C, we adjusted the number of PARACLU, RECLU and CAGER predictions to match the 65,141 FANTOM CAGE peaks in H9 cells, to keep the evaluation consistent. Each algorithm provides a different number of predictions for every parameter combination. Therefore, for a given combination, we overlapped the 65,141 peaks with the set of predictions. Peaks that overlapped with at least one prediction were considered positives for the corresponding parameter combination, while the remaining peaks were considered negatives. With this approach, all algorithms could be evaluated on the same set of predictions, which is a critical feature for an annotation-based comparison.

For TOMETOOLS, we downloaded TSS_human bed formatted file (hg19 genome assembly) from http://fantom.gsc.riken.jp/5/datafiles/phase1.3/extra/TSS_classifier. This file contains 1,048,124 CAGE enriched loci, as identified by all profiled cell types in FANTOM, scored by TOMETOOLS. The coordinates were converted to the hg38 genome assembly using liftover software from UCSC. In the TSS_human file, there is no information related to which CAGE peaks are expressed in H9 or K562 cells. To keep the comparison consistent and in the context of these two cell types, we overlapped the regions in TSS_human with the two files containing the H9 (N = 65,141) and K562 (N = 47,377) CAGE peaks. This way, we generated TOMETOOLS predictions in H9 and K562 cells (Supplementary Table 1). The 0.228 TOMETOOLS strict score cutoff for creating positive and negative predictions was selected based on the documentation of the algorithm. We chose the strict threshold over the loose to match the strict threshold selection for ADAPT-CAGE. For the comparisons that required multiple score cutoffs, the range of applied thresholds is shown in Supplementary Tables 4.

Size distributions of CAGE tag-clusters provided by each algorithm are presented in Supplementary Fig. 7 based on the H9 cells sample. ADAPT-CAGE tag-clusters were generated by the first pre-classification step of the algorithm (tag-cluster generator) with a distance parameter value (for aggregating tags into clusters) of 10, 25 and 50 bp. Tag-clusters from RECLU, PARACLU, CAGER and TOMETOOLS, using default setting, are also shown. Note that in all main and supplementary evaluation figures, ADAPT-CAGE has been applied on CAGE tag-clusters as reported by FANTOM.

The repeated application of existing implementations using different parameters, as described above, leads to the creation of overlapping sets of predictions based on a gradual increase in the certainty related to the quality (or robustness) of provided results. This approach, combined with the usage of multiple ADAPT-CAGE and TOMETOOLS score cutoffs, practically corresponds to the selection of different percentiles from the total amount of predictions of each algorithm (i.e. the top 10%, top 20% etc). Additionally, since existing implementations are not based on Machine Learning, the approach of multiple runs based on different parameters, also corresponds to the strategy documented in FANTOM repository that selects CAGE peaks with respect to read count associated thresholding. This is also evident from the certain parameters that are supported by these algorithms that correspond to either raw or normalized read-count cutoffs.

## Supplementary information

Supplementary Information.

Supplementary table 1.

Supplementary table 2.

Supplementary table 3.

Supplementary table 4.

Supplementary table 5.

Supplementary table 6.

Supplementary table 7.

## Data Availability

ADAPT-CAGE is an open source computational framework freely accessible in https://gitlab.com/dianalab/adapt-cage.
