# Supplementary material for: Solving the transcription start site identification problem with ADAPT-CAGE: a Machine Learning algorithm for the analysis of CAGE data
Source: Sci Rep. 2020 Jan 21;10:877. doi: 10.1038/s41598-020-57811-3 (PMC6972925; doi:10.1038/s41598-020-57811-3)
Supplement: Supplementary file 1 — Supplementary Information. [file 41598_2020_57811_MOESM1_ESM.pdf]

Supplementary Information

Solving the transcription start site identification problem with ADAPT-CAGE: a Machine Learning algorithm for the analysis of CAGE data

Georgios K Georgakilas[1,2,3,†,*], Nikos Perdikopanis[1,2,4,†] and Artemis Hatzigeorgiou[1,2,*].

[1] Hellenic Pasteur Institute, Athens, 11521, Greece.
[2] Department of Electrical and Computer Engineering, University of Thessaly, Volos, Greece.
[3] Central European Institute of Technology, Masaryk University, Kamenice 735/5, 62500 Brno, Czech Republic.
[4] Department of Informatics and Telecommunications, National and Kapodistrian University of Athens, Athens, Greece.

† Equal contribution.

*To whom correspondence should be addressed. Tel: +30 24210 74758; Fax: +30 24210 74997; Email: arhatzig@e-ce.uth.gr. Correspondence may also be addressed to George Georgakilas. Tel: +30 24210 74758; Fax: +30 24210 74997; Email:georgios.georgakilas@ceitec.muni.cz.

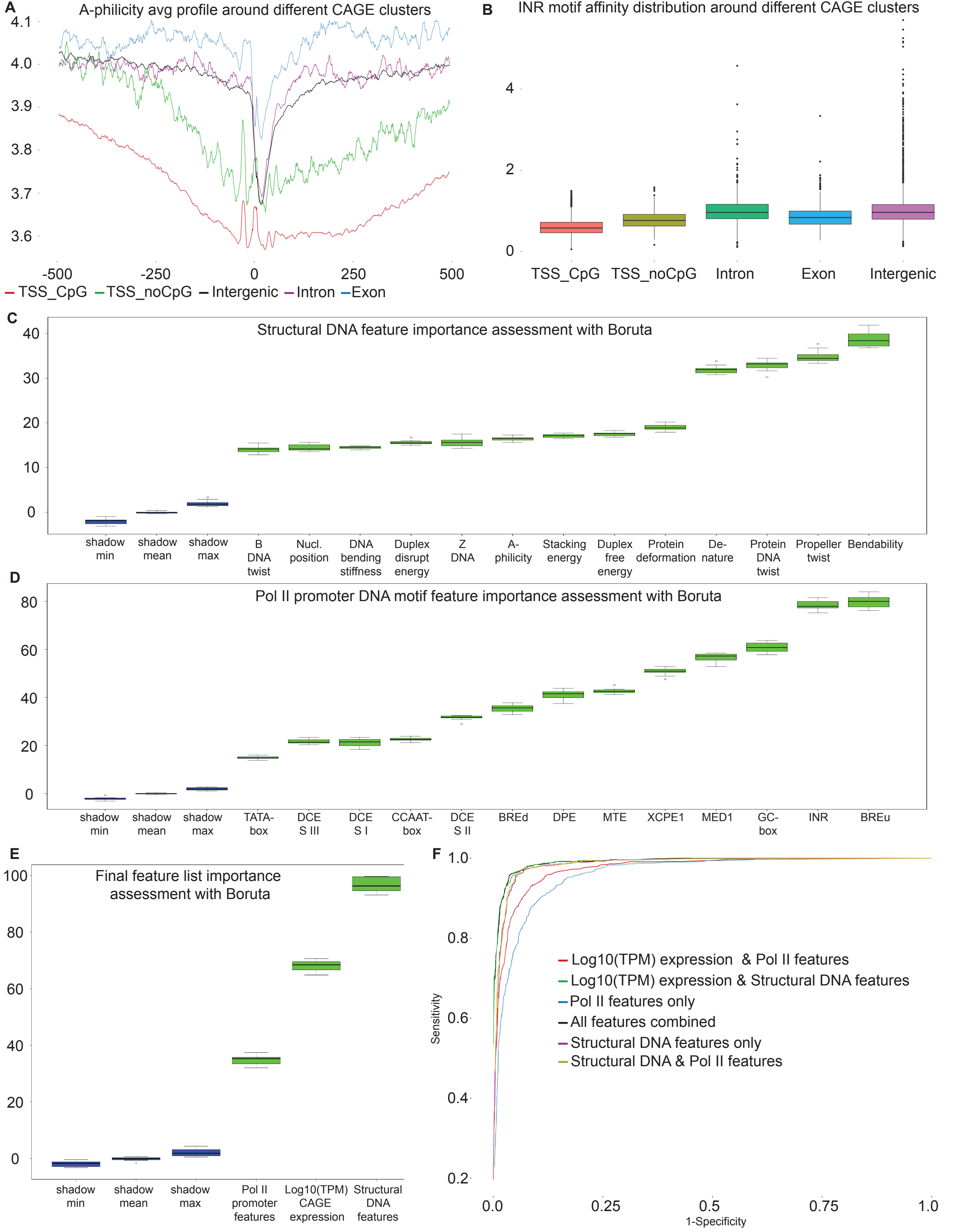

A
A-philicity avg profile around different CAGE clusters
4.1
4.0
3.9
3.8
3.7
3.6
-500
-250
0
250
500
TSS_CpG
TSS_noCpG
Intergenic
Intron
Exon
B
INR motif affinity distribution around different CAGE clusters
4
2
0
TSS_CpG
TSS_noCpG
Intron
Exon
Intergenic
C
Structural DNA feature importance assessment with Boruta
40
30
20
10
0
shadow min
shadow mean
shadow max
B DNA twist
Nucl. position
DNA bending stiffness
Duplex disrupt energy
Z DNA
A-philicity
Stacking energy
Duplex free energy
Protein deformation
De-nature
Protein DNA twist
Propeller twist
Bendability
D
Pol II promoter DNA motif feature importance assessment with Boruta
80
60
40
20
0
shadow min
shadow mean
shadow max
TATA-box
DCE S III
DCE S I
CCAAT-box
DCE S II
BREd
DPE
MTE
XCPE1
MED1
GC-box
INR
BREu
E
Final feature list importance assessment with Boruta
100
80
60
40
20
0
shadow min
shadow mean
shadow max
Pol II promoter features
Log10(TPM) CAGE expression
Structural DNA features
F
1.0
0.8
0.6
0.4
0.2
Sensitivity
0.0
0.25
0.5
0.75
1.0
1-Specificity
Log10(TPM) expression & Pol II features
Log10(TPM) expression & Structural DNA features
Pol II features only
All features combined
Structural DNA features only
Structural DNA & Pol II features

Supplementary Figure 1. Feature importance assessment and visualization.

Representative examples of each feature category; structure (i.e. A-philicity) in (A) and promoter-associated motifs (i.e.INR) in (B). The distribution of A-philicity is depicted as an average profile and the distribution of INR affinity as boxplots around CAGE tag-clusters located in different regions of the genome. C) Assessment of each structural feature's importance with the Boruta R package. This corresponds to the second layer of training, as depicted in Figure 1B, for the SGB model that combines the output of the structural feature SVM models. D) Assessment of each promoter-associated feature's importance. This corresponds to the second layer of training, as depicted in Figure 1B, for the SGB model that combines the Polymerase II motif affinity values. E) Assessment of the importance of the final structural and promoter feature models, as well as the log10(TPM) expression level for the classification task. This corresponds to the last layer of training, as depicted in Figure 1B, for the SGB model that combines the output of the second layer SGB models and the normalized CAGE expression value. F) ROC curves of models based on different feature combinations performance on the test set.

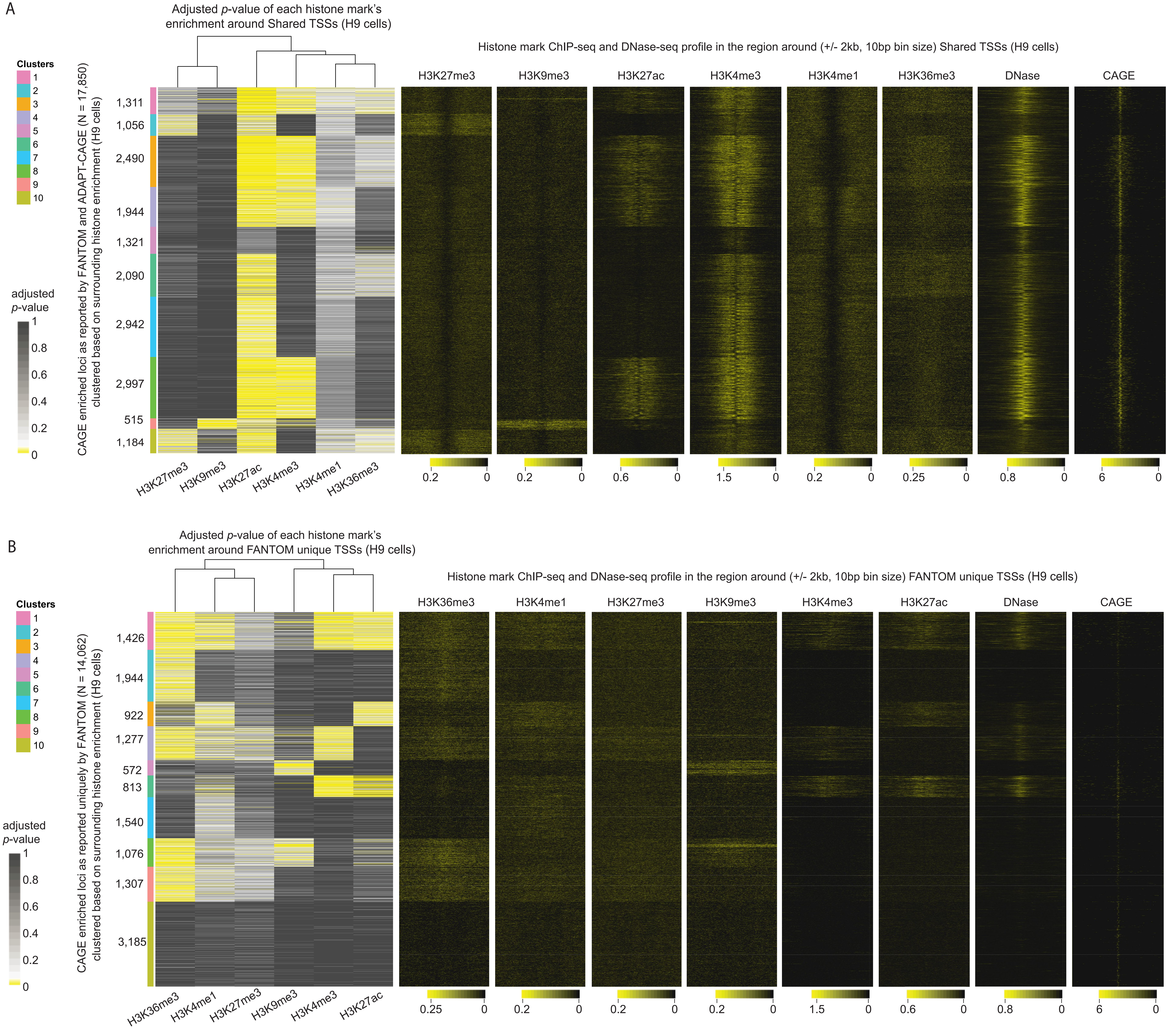
A
Adjusted p-value of each histone mark's enrichment around Shared TSSs (H9 cells)
Histone mark ChIP-seq and DNase-seq profile in the region around (+/- 2kb, 10bp bin size) Shared TSSs (H9 cells)
Clusters
1
2
3
4
5
6
7
8
9
10
adjusted p-value
1
0.8
0.6
0.4
0.2
0
CAGE enriched loci as reported by FANTOM and ADAPT-CAGE (N = 17,850) clustered based on surrounding histone enrichment (H9 cells)
1,311
1,056
2,490
1,944
1,321
2,090
2,942
2,997
515
1,184
H3K27me3
H3K9me3
H3K27ac
H3K4me3
H3K4me1
H3K36me3
H3K27me3
H3K9me3
H3K27ac
H3K4me3
H3K4me1
H3K36me3
DNase
CAGE
0.2
0
0.2
0
0.6
0
1.5
0
0.2
0
0.25
0
0.8
0
6
0
B
Adjusted p-value of each histone mark's enrichment around FANTOM unique TSSs (H9 cells)
Histone mark ChIP-seq and DNase-seq profile in the region around (+/- 2kb, 10bp bin size) FANTOM unique TSSs (H9 cells)
Clusters
1
2
3
4
5
6
7
8
9
10
adjusted p-value
1
0.8
0.6
0.4
0.2
0
CAGE enriched loci as reported uniquely by FANTOM (N = 14,062) clustered based on surrounding histone enrichment (H9 cells)
1,426
1,944
922
1,277
572
813
1,540
1,076
1,307
3,185
H3K36me3
H3K4me1
H3K27me3
H3K9me3
H3K4me3
H3K27ac
H3K36me3
H3K4me1
H3K27me3
H3K9me3
H3K4me3
H3K27ac
DNase
CAGE
0.25
0
0.2
0
0.2
0
0.2
0
1.5
0
0.6
0
0.8
0
6
0

Supplementary Figure 2. Genome-wide assessment of the differences in the chromatin activity environment surrounding CAGE enriched loci in H9 cells after applying ADAPT-CAGE (0.5 score cutoff).

Clustering of CAGE tag-clusters positively (A) and negatively (B) scored for 0.5 score cutoff by ADAPT-CAGE, based on the surrounding enrichment of six histone marks' signal. The normalized profile of all histone marks' as well as DNase-Seq and CAGE signal is added as a visual aid.

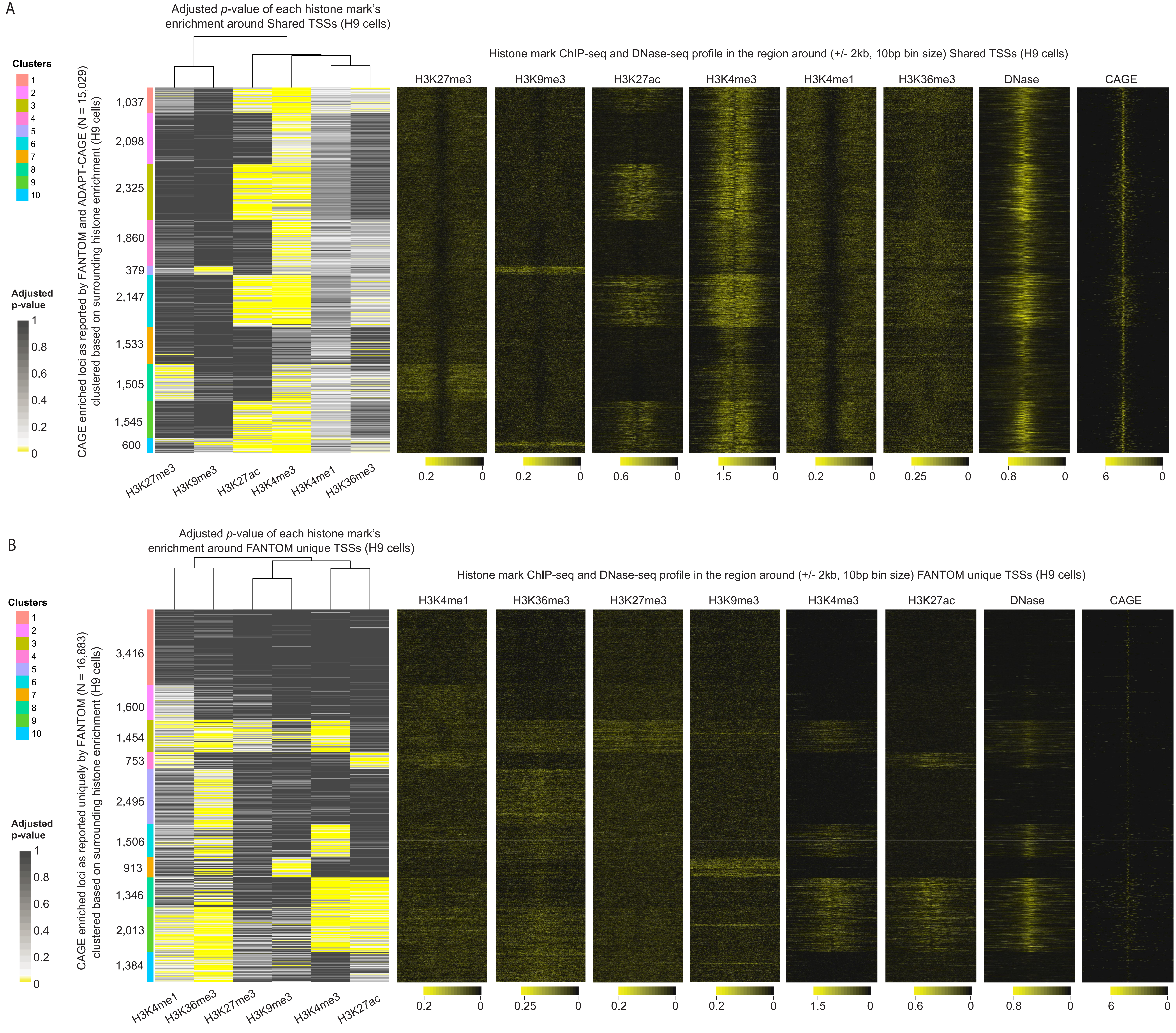

A
Adjusted p-value of each histone mark's enrichment around Shared TSSs (H9 cells)
Histone mark ChIP-seq and DNase-seq profile in the region around (+/- 2kb, 10bp bin size) Shared TSSs (H9 cells)
Clusters
1
2
3
4
5
6
7
8
9
10
Adjusted p-value
1
0.8
0.6
0.4
0.2
0
CAGE enriched loci as reported by FANTOM and ADAPT-CAGE (N = 15,029) clustered based on surrounding histone enrichment (H9 cells)
1,037
2,098
2,325
1,860
379
2,147
1,533
1,505
1,545
600
H3K27me3
H3K9me3
H3K27ac
H3K4me3
H3K4me1
H3K36me3
DNase
CAGE
0.2
0
0.2
0
0.6
0
1.5
0
0.2
0
0.25
0
0.8
0
6
0
B
Adjusted p-value of each histone mark's enrichment around FANTOM unique TSSs (H9 cells)
Histone mark ChIP-seq and DNase-seq profile in the region around (+/- 2kb, 10bp bin size) FANTOM unique TSSs (H9 cells)
Clusters
1
2
3
4
5
6
7
8
9
10
Adjusted p-value
1
0.8
0.6
0.4
0.2
0
CAGE enriched loci as reported uniquely by FANTOM (N = 16,883) clustered based on surrounding histone enrichment (H9 cells)
3,416
1,600
1,454
753
2,495
1,506
913
1,346
2,013
1,384
H3K4me1
H3K36me3
H3K27me3
H3K9me3
H3K4me3
H3K27ac
DNase
CAGE
0.2
0
0.25
0
0.2
0
0.2
0
1.5
0
0.6
0
0.8
0
6
0

Supplementary Figure 3. Genome-wide assessment of the differences in the chromatin activity environment surrounding CAGE enriched loci in H9 cells after applying ADAPT-CAGE (0.9 score cutoff).

Clustering of CAGE tag-clusters positively (A) and negatively (B) scored for 0.9 score cutoff by ADAPT-CAGE, based on the surrounding enrichment of six histone marks' signal. The normalized profile of all histone marks' as well as DNase-Seq and CAGE signal is added as a visual aid.

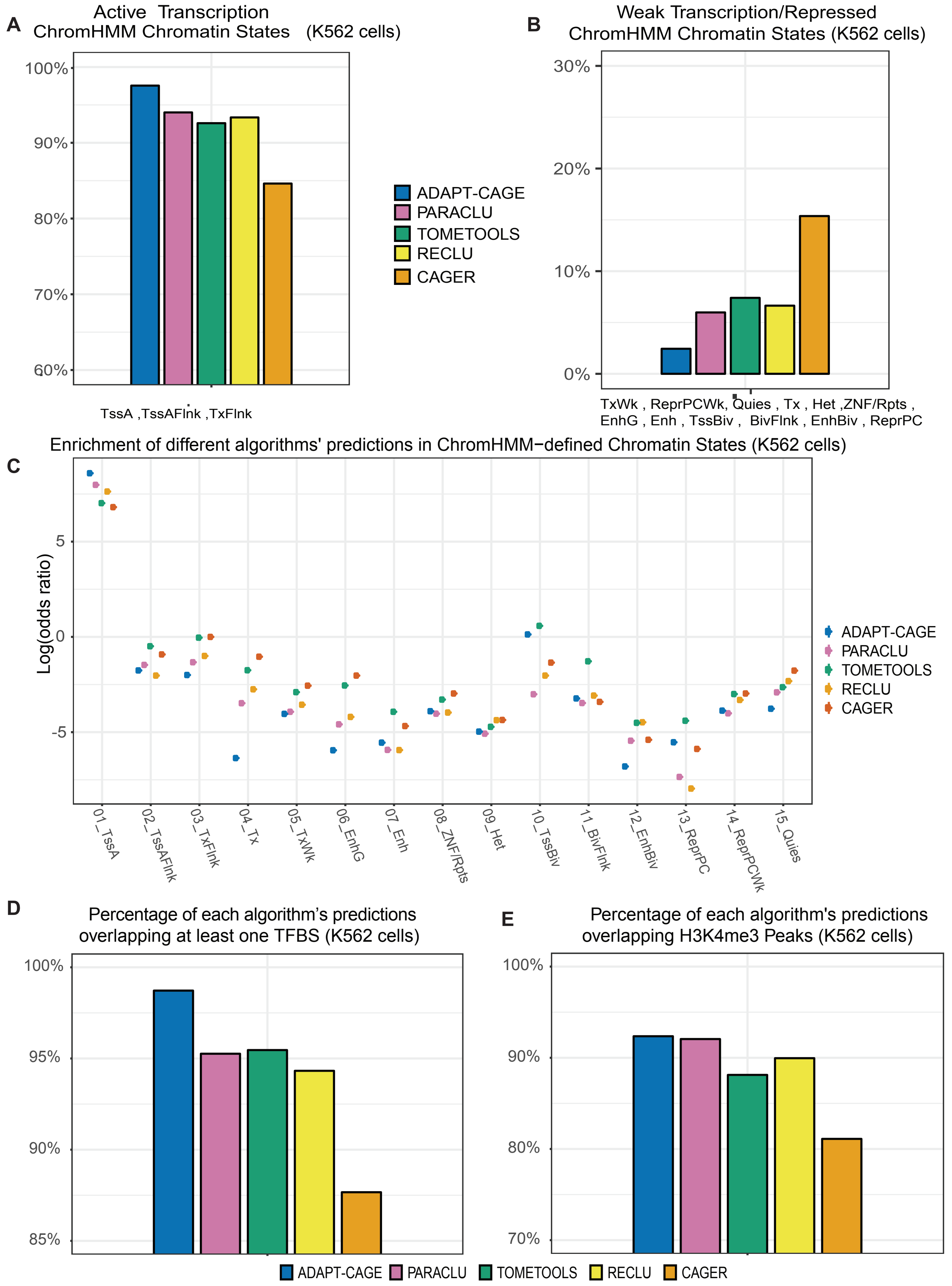

A
Active Transcription
ChromHMM Chromatin States (K562 cells)
100%
90%
80%
70%
60%
TssA ,TssAFlnk ,TxFlnk
ADAPT-CAGE
PARACLU
TOMETOOLS
RECLU
CAGER
B
Weak Transcription/Repressed
ChromHMM Chromatin States (K562 cells)
30%
20%
10%
0%
TxWk , ReprPCWk, Quies , Tx , Het ,ZNF/Rpts ,
EnhG , Enh , TssBiv , BivFlnk , EnhBiv , ReprPC
C
Enrichment of different algorithms' predictions in ChromHMM−defined Chromatin States (K562 cells)
Log(odds ratio)
5
0
-5
01_TssA
02_TssAFlnk
03_TxFlnk
04_Tx
05_TxWk
06_EnhG
07_Enh
08_ZNF/Rpts
09_Het
10_TssBiv
11_BivFlnk
12_EnhBiv
13_ReprPC
14_ReprPCWk
15_Quies
ADAPT-CAGE
PARACLU
TOMETOOLS
RECLU
CAGER
D
Percentage of each algorithm’s predictions
overlapping at least one TFBS (K562 cells)
100%
95%
90%
85%
E
Percentage of each algorithm's predictions
overlapping H3K4me3 Peaks (K562 cells)
100%
90%
80%
70%
ADAPT-CAGE
PARACLU
TOMETOOLS
RECLU
CAGER

Supplementary Figure 4. Evaluating algorithms' performance on experimental data in K562 cells.

Percentage of each algorithm's predictions uniquely overlapping ChromHMM-derived chromatin states based on the core-15 model in K562 cells. Chromatin states were aggregated in two groups according to levels of chromatin/transcriptional activity. Active (A) and weaker (B) transcription states. C) Enrichment (odds ratio in logarithmic scale) of algorithms' prediction in all ChromHMM-derived chromatin states (TssA and Quies). Percentage of each algorithm's predictions overlapping at least one transcription factor binding site (D) and H3K4me3 (E) ChIP-Seq derived peaks.

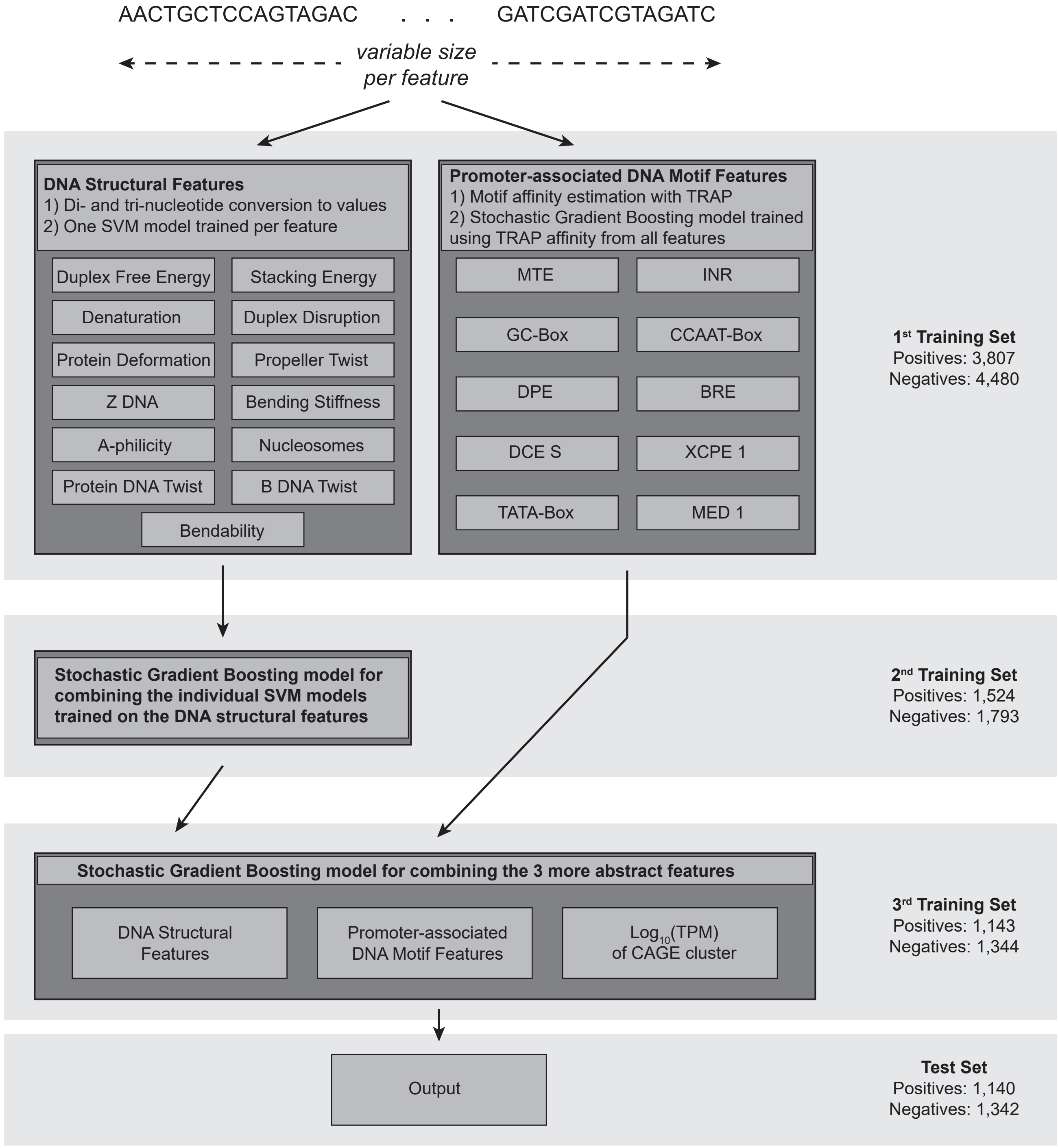
AACTGCTCCAGTAGAC . . . GATCGATCGTAGATC
variable size per feature
DNA Structural Features
1) Di- and tri-nucleotide conversion to values
2) One SVM model trained per feature
Duplex Free Energy
Stacking Energy
Denaturation
Duplex Disruption
Protein Deformation
Propeller Twist
Z DNA
Bending Stiffness
A-philicity
Nucleosomes
Protein DNA Twist
B DNA Twist
Bendability
Promoter-associated DNA Motif Features
1) Motif affinity estimation with TRAP
2) Stochastic Gradient Boosting model trained using TRAP affinity from all features
MTE
INR
GC-Box
CCAAT-Box
DPE
BRE
DCE S
XCPE 1
TATA-Box
MED 1
1st Training Set
Positives: 3,807
Negatives: 4,480
Stochastic Gradient Boosting model for combining the individual SVM models trained on the DNA structural features
2nd Training Set
Positives: 1,524
Negatives: 1,793
Stochastic Gradient Boosting model for combining the 3 more abstract features
DNA Structural Features
Promoter-associated DNA Motif Features
Log10(TPM) of CAGE cluster
3rd Training Set
Positives: 1,143
Negatives: 1,344
Output
Test Set
Positives: 1,140
Negatives: 1,342

Supplementary Figure 5. Overview of ADAPT-CAGE training process based on CAGE samples from H1 cells.

CAGE tag-clusters with less than 1 TPM expression level were removed. From the remaining regions, tag-clusters overlapping H3K4me3 and Polymerase II ChIP-Seq derived peaks, located on annotated promoters consisted the positive set. Tag-clusters overlapping intronic and exonic regions or located in intergenic space, but not overlapping H3K4me3 or Polymerase II peaks, consisted the negative set. Both negative and positive sets were subsequently split into 4 sets, 3 of which were kept for training the different layers of the model and 1 for testing the final performance. Raw sequences were scanned to calculate the distribution of each structural feature as well as the affinity of every promoter associated JASPAR motif. Each structural feature was responsible for training its own SVM model while the promoter motifs were combined into an SGB model using the 1st training set. The second training set was used to train the SGB that aggregates the structural DNA SVM models, while the third one was used to train the SGB model that combines the structural and promoter motif features with the log10(TPM) value of CAGE tag-clusters.

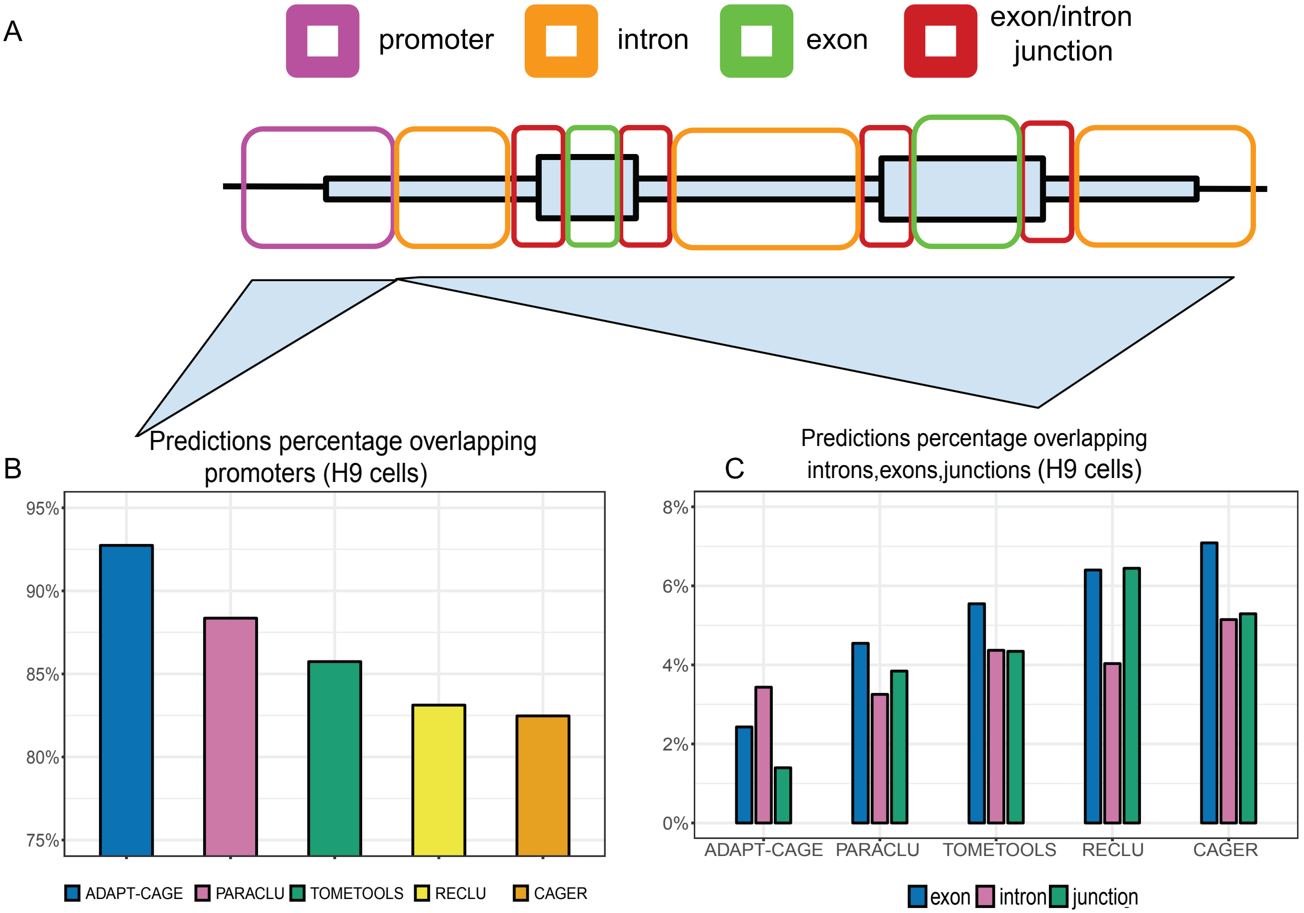
A
promoter
intron
exon
exon/intron junction
B
Predictions percentage overlapping promoters (H9 cells)
95%
90%
85%
80%
75%
ADAPT-CAGE
PARACLU
TOMETOOLS
RECLU
CAGER
C
Predictions percentage overlapping introns,exons,junctions (H9 cells)
8%
6%
4%
2%
0%
ADAPT-CAGE
PARACLU
TOMETOOLS
RECLU
CAGER
exon
intron
junction

Supplementary Figure 6. Comparison of algorithms' performance based on segmentation of genic regions.

A) Segmentation of genic regions into different types of annotation such as promoters, introns, exons and exon/intron junctions. Percentage of algorithms' prediction overlapping promoters (B) and other genic types of annotation (C).

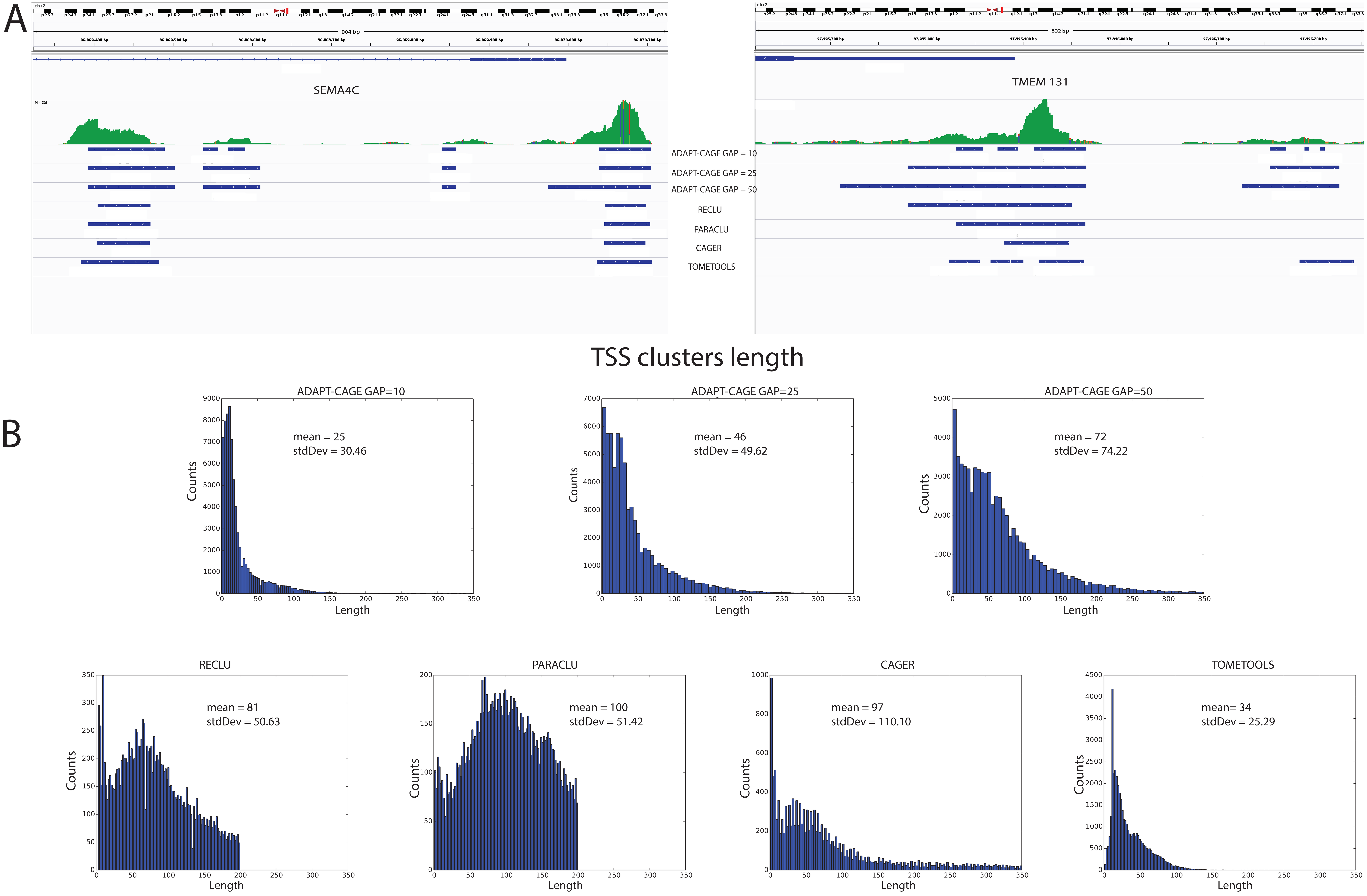
A
SEMA4C
804 bp
TMEM 131
632 bp
ADAPT-CAGE GAP = 10
ADAPT-CAGE GAP = 25
ADAPT-CAGE GAP = 50
RECLU
PARACLU
CAGER
TOMETOOLS
B
TSS clusters length
ADAPT-CAGE GAP=10
mean = 25
stdDev = 30.46
ADAPT-CAGE GAP=25
mean = 46
stdDev = 49.62
ADAPT-CAGE GAP=50
mean = 72
stdDev = 74.22
RECLU
mean = 81
stdDev = 50.63
PARACLU
mean = 100
stdDev = 51.42
CAGER
mean = 97
stdDev = 110.10
TOMETOOLS
mean= 34
stdDev = 25.29
Counts
Length

Supplementary Figure 7. Genome browser instances of CAGE tag-clusters provided by all algorithms and size distribution of CAGE tag-clusters, based on the H9 cells sample.

A) Genome browser example of the two gene loci presented in Figure 2, SEMA4C and TMEM131. From top to bottom, the raw CAGE signal is shown in green color, ADAPT-CAGE tag-clusters with a distance parameter value (for aggregating tags into clusters) of 10, 25 and 50bp, RECLU, PARACLU, CAGER and TOMETOOLS tag-clusters in blue. For ADAPT-CAGE, only the first pre-classification step of the algorithm (the CAGE tag-cluster assembler) was used to generate the tag-clusters depicted here, in contrast to the other algorithms. This is the reason why, for example, the CAGE enriched region on the right side of TMEM131 panel is not supported by RECLU, PARACLU and CAGER, probably due to their default expression level parameter cutoff. B) Size distribution of CAGE tag-clusters provided by ADAPT-CAGE with three different distance parameter values (10, 25 and 50bp) for aggregating reads into clusters, RECLU, PARACLU, CAGER and TOMETOOLS, on default settings.
